# Possession and Applicability of Signature Character Strengths: What Is Essential for Well-Being, Work Engagement, and Burnout?

**DOI:** 10.1007/s11482-018-9699-8

**Published:** 2019-01-13

**Authors:** Alexandra Huber, Cornelia Strecker, Melanie Hausler, Timo Kachel, Thomas Höge, Stefan Höfer

**Affiliations:** 1 Department of Medical Psychology, Medical University of Innsbruck, Innsbruck, Austria; 2 Institute of Psychology, University of Innsbruck, Innsbruck, Austria

**Keywords:** Signature character strengths, Applicability, Physicians, Well-being, Work engagement, Burnout

## Abstract

Signature character strengths can foster health-related outcomes in work and private life, thus being particularly important for endangered occupational groups like physicians. However, situational circumstances need to allow character strengths demonstration (applicability) first to enable their application. Therefore, this study addresses the role of (1) applicability of signature character strengths in work and private life beyond their possession and (2) relationships with well-being, work engagement, and burnout dimensions (emotional exhaustion, depersonalization, and reduced personal accomplishment). Hospital physicians (*N* = 274) completed an online survey examining their signature character strengths and applicability, well-being, work engagement, and burnout dimensions. The top-five individual signature character strengths were fairness, honesty, judgment, kindness, and love. Hierarchical multiple linear regressions revealed that the possession as well as the applicability of signature character strengths was important in work and private life, but to different degrees. Possessing fairness, honesty, or kindness indicated significant positive relations with subjective well-being, whereas judgment and kindness seemed to negatively interact with reduced personal accomplishment. Hospital physicians’ applicability of fairness, honesty, judgment, and love was particularly essential for their psychological well-being and work engagement, whereas the applicability of fairness (reduced personal accomplishment) and judgment (emotional exhaustion, depersonalization) at work interacted negatively with the respective outcomes. Therefore, creating awareness for individual signature character strengths as well as providing applicability in hospitals and private life could be a promising approach to improve physicians’ well-being and consequently patient care as well as the performance of the health-care system in general.

## Introduction

The research area of positive psychology emerged in the late 1990s and deals with factors that make life most worth living to fulfill individual potentials by fostering human functioning (Seligman and Csikszentmihalyi 2000). In positive psychology three main scopes are relevant: a) positive subjective experiences (e.g., happiness/satisfaction), b) positive individual traits (e.g., character strengths), and c) positive institutions (e.g., families/workplaces; Peterson 2006; Seligman and Csikszentmihalyi 2000). Beside the importance of possessing character strengths, their demonstration needs to be allowed or called (applicability) in particular by positive institutions to enable application in various life domains and foster deepened positive experiences (Govindji and Linley 2007; Peterson 2006; Rath 2007). Therefore, this analysis addresses the role of applicability of character strengths in work and private life beyond their possession and relationships with well-being, work engagement, and burnout.

### Character Strengths

Peterson and Seligman (2004) introduced the Values in Action (VIA) classification to describe the good human character, representing 24 character strengths assigned to six different virtues (courage, humanity, justice, temperance, transcendence, wisdom) which have been theoretically considered important across many religions and cultures, going back to the Noble Eightfold Path (Buddhism, fifth century B.C.) and the Ten Commandments (first century B.C.). Character strengths are conceptualized as positive, stable and moral traits and their possession can be measured with the VIA-Inventory of Strengths (VIA-IS; Peterson and Park 2009; Peterson and Seligman 2004). Possession here means to have character strengths at least to a certain degree (see Harzer and Ruch 2013). Moreover, Peterson and Seligman claimed that everybody has about three to seven character strengths which are typical of an individual, akin to what Allport identified 1961 as personal traits (see Peterson and Seligman 2004), the so-called ‘signature character strengths’. When people own, celebrate and frequently apply them, they are related to e.g., feelings of authenticity (‘this is the real me’) and excitement while displaying, people yearn to act in accordance with the strength, continuously learn new ways to enact the strength, and feel intrinsically motivated to apply them (Peterson and Seligman 2004). Therefore, they support peoples’ accomplishment or engagement (e.g., creation and pursuit of projects that revolve around the strength) and positive emotions (e.g., invigoration rather than exhaustion when applying strengths). But to be able to apply these individual (signature) character strengths and show trait-related behavior, situational circumstances in work or private life need to be conducive (Saucier et al. 2007) or in other words, offer applicability. Therefore, the ‘Applicability of Character Strengths Rating Scales’ (ACS-RS) aim to measure the extent to which each of the 24 character strengths of the VIA-IS is applicable (e.g., ‘demanded’, ‘helpful’ or ‘important’) in work and private life (Harzer and Ruch 2013).

Theoretical research perspectives have focused on both, possessing and applying character strengths. Empirically, studies have tended to concentrate more on their possession to a certain degree than on their applicability or application (e.g., Park et al. 2004; Peterson et al. 2007) whereas preliminary evidence indicated that particularly the latter substantially contributes to well-being (Gander et al. 2013; Govindji and Linley 2007; Rath 2007). This augmented focus on the possession of character strengths might be due to the late absence of psychometrically adequate scales (e.g., Harzer and Ruch 2013; Littman-Ovadia and Steger 2010) or to the circumstance that the VIA-IS measures possession of character strengths with items already including behavior patterns and applicability/application to some extent (e.g., ‘When the topic calls for it, I can be a highly rational thinker’, ‘Everyone’s rights are equally important to me’, ‘I really enjoy doing small favors for friends’) making it even harder to exactly differentiate between possessing or applying character strengths.

### Positive Experiences in Private and Work Life

#### Well-Being

Research on well-being can be differentiated in two traditions (Deci and Ryan 2008). In the hedonistic tradition the focus is on happiness, generally defined by life satisfaction, the presence of positive and the absence of negative affect (subjective well-being; Diener 1984) concerning different life domains (work, family, leisure, health, finances, self, and one’s group; Diener et al. 1999). In the eudaimonic tradition the focus is on living one’s life in a deeply satisfying way and countering existential challenges in daily life (psychological well-being) concerning aspects like autonomy, engagement, mastery, meaning, optimism, and relationships (e.g., Su et al. 2014). Although concepts of subjective and psychological well-being are related and interdependent, they are empirically distinct (Ring et al. 2007).

The 24 VIA-character strengths have been repeatedly analyzed in relation to well-being outcomes. The most strongly related character strengths to life satisfaction (as being part of subjective well-being) were curiosity (*r* = .38–.39), gratitude (*r* = .37–.43), hope (*r* = .53–.59), love (*r* = .44–.46), and zest (*r* = .45–.53; e.g., Buschor et al. 2013; Harzer 2016; Park et al. 2004) as well as to subjective well-being in total (curiosity: *r* = .39 to hope: *r* = .67; Hausler et al. 2017a). These character strengths are the so-called ‘happiness strengths’. The application of e.g., hope and zest predicted subjective well-being in two UK student samples and correlated positively with self-esteem (Proctor et al. 2011) and positive affect (Ouweneel et al. 2014). Concerning psychological well-being there is less research, but first results suggested that possessing happiness strengths is important as well (love: *r* = .38 to hope: *r* = .59; Hausler et al. 2017a). Moreover, its aspects seem to be related to specific character strengths (*r* ≥ .40): autonomy to bravery, curiosity, honesty, hope, perspective, and zest; engagement to curiosity, gratitude, humor, perseverance, and zest; mastery to creativity, curiosity, fairness, honesty, hope, perseverance, and zest; meaning to bravery, curiosity, gratitude, hope, leadership, love, perspective, spirituality, and zest; optimism to hope and zest; relationships to hope, love, social intelligence, and zest (Harzer 2016; Hausler et al. 2017a; Peterson et al. 2007). Overall, more subjective/psychological wellbeing can be achieved by (more frequently) applying character strengths (e.g., Allan and Duffy 2014; Douglass and Duffy 2014; Gander et al. 2013; Lyubomirsky et al. 2005; Proyer et al. 2014; Seligman et al. 2005).

#### Work Engagement and Burnout

Task- and organization-related working conditions (e.g., demands, resources, and stressors) can affect employees’ healthiness, well-being, and motivation. Their outcomes (e.g., work engagement, burnout) can occur simultaneously in positive or negative ways (Glaser and Seubert 2014; Schaufeli et al. 2009). In particular, work engagement can contribute positively to occupational well-being (besides job motivation, organizational commitment, and work satisfaction) and is defined as positive, fulfilling work-related motivational state of mind characterized by vigor, dedication, and absorption (Schaufeli et al. 2002). It is inspired by positive psychology and characterizes a view on work-related well-being that takes personality- and health-promoting effects of work into account (Schaufeli et al. 2002), conceptualized as positive counterpart of job burnout. Burnout is usually defined as a syndrome comprising three dimensions: emotional exhaustion (depleted emotional and internal resources, feelings to not have anything more to give to the job), depersonalization (attempt to distance oneself from the job, feelings of increasing cynicism about the value of work, actively starting to ignore positive aspects of the job), and reduced personal accomplishment (feelings of much less effectiveness in the job, performance decreases; Maslach et al. 2001). Emotional exhaustion is strongly related to physical health outcomes (e.g., diabetes, infections, and cardiovascular diseases) and mental illness (Shirom et al. 2005) as well as high depersonalization (Leiter and Maslach 2016). However, the absence of burnout symptoms does not automatically mean that a person is engaged in work tasks.

First results indicated that the possession of happiness strengths is related to occupational well-being across a range of different professions (e.g., love: increased job satisfaction with work that explicitly involves other people; zest: work as calling; Peterson and Park 2006; Peterson et al. 2007). Moreover, the application of character strengths at work is related to various positive experiences (e.g., pleasure, work engagement, meaning) and job satisfaction (Harzer and Ruch 2012, 2013; Littman-Ovadia and Steger 2010; Peterson and Park 2011; Seligman 2011) as well as behavioral outcomes (e.g., perseverance; Littman-Ovadia and Lavy 2016). Littman-Ovadia et al. (2017) described in a study, distinguishing between signature character strengths and happiness strengths, that using the former contributed more to behavioral outcomes (job performance *r* = .23, organizational citizenship behavior *r* = .38, and counterproductive work behavior *r* = −.22) whereas happiness strengths were more related to psycho-emotional outcomes (work engagement *r* = .65, job satisfaction *r* = .54, and meaningfulness *r* = .59). Moreover, one study revealed the effect of work engagement mediating the relation of character strengths application and productivity, organizational citizenship behavior, and job satisfaction (Lavy and Littman-Ovadia 2017). Concerning burnout, one study examined work-related patterns and revealed that the resigned type (burnout working behavior; positively associated with the burnout dimensions) scored significantly lower in all character strengths than the healthy-ambitious type (actively coping, experiencing social support, being able to keep emotional distance from work, and to be satisfied with work and life in general); particularly large effect sizes were found in terms of hope, perseverance, and zest (η2 = .40–.50; Gander et al. 2012). Significant indirect effects via emotional exhaustion (being one key dimension of burnout according to Maslach et al. 2001) were found recently concerning the link: ‘applicability of character strengths’ to ‘subjective/psychological well-being, mental and physical health’ in medical students (β_indirect_ = .05–.15) and resident physicians (excl. Mental health, β_indirect_ = .05–.09; Hausler et al. 2017b).

### Physicians in Positive Institutions?

Physicians belong to an endangered occupational group exposed to tremendous work demands and stressors (e.g., workload, time pressure, emotional interactions, cognitive demands) and when they feel unwell, the performance of health-care systems as well as patient care can be impaired (e.g., Wallace et al. 2009). A Canadian study revealed that 64% of physicians feel that their workload is too heavy and 48% experienced an increased workload in the past year leading to more absenteeism, job turnover, and earlier retirement (Canadian Medical Association 2003). When physicians frequently have work shifts lasting longer than 24 h, the resulting fatigue is associated with negative personal and professional consequences: they have an increased burnout risk (resident physicians 60%; physicians 51%; Dyrbye et al. 2014), report significantly more failures of attention, and serious medical errors than those with shorter shifts (Landrigan et al. 2004; Lockley et al. 2004). Depression, substance abuse (antipsychotics, benzodiazepines, barbiturates), and suicide occurred as well above-average in physicians compared to the general population (Gold et al. 2013). Burnout and depression had a particular effect on medical errors (two to three times increased probability of reporting an error at least monthly or weekly) and moreover, rapid and recent changes to the practice of medicine (e.g., increased patient-care demands, wages, growing bureaucracy, and accountability) seem to be potential threats to physicians’ well-being (Wallace et al. 2009). In Germany and Austria researchers found similar prevalences of burnout (up to 50%) and depression (10%) in local physicians (Heinke et al. 2011; Weigl et al. 2012; Wurm et al. 2016) corresponding to international findings. Therefore, it is questionable to what extent hospitals are positive institutions which allow or call for the demonstration of character strengths.

### Aims of this Analysis

According to research so far, empirical evidence is lacking whether fostering applicability of character strengths in hospitals could be a promising approach to improve physicians’ well-being. The medical occupation itself is already challenging and when working in conditions as outlined above, approaches to foster physicians’ resources such as investigations of character strengths deployment are needed. Therefore, two aims emerged. (1) As the VIA-IS measures the possession of character strengths with items already including some behavior patterns and applicability/application, this questionnaire was compared with the ACS-RS to test if the two instruments measure theoretically distinct constructs or not. If results indicate diversity, (2) the applicability of signature character strengths in work and private life will be examined in terms of whether there is a potential to increase well-being and work engagement and prevent burnout of hospital physicians beyond the possession of signature character strengths. Therefore, the five most frequently reported individual signature character strengths will be examined.

## Methods

### Sample and Procedure

The study was conducted in two hospitals of a regional network including a large university hospital in Austria from 2015 to 2017. After ethic commission and institutional review board approval was given, hospital physicians completed an online survey. Offered incentives were vouchers (10 vouchers for a brunch at 50 Euros each) in a raffle. Data collection was part of a larger research project on health and well-being of medical students and hospital physicians funded by the Austrian Science Fund. A total of *N* = 274 physicians participated. About 62% of participants were female, about 38% were male. The mean age was 34.2 years (SD = 8.1, range = 24–64 years). A large majority of *N* = 215 (78.5%) were resident physicians in training, *N* = 59 (21.5%) were senior medical specialists. The physicians worked in 14 different medical disciplines.

### Measures

#### Character Strengths

For measuring individual character strengths, the German 120-item version of the VIA-IS (VIA-120) was used (Höfer et al. 2018, in this special issue; Littman-Ovadia 2015; original: VIA Institute on Character 2014). Psychometric properties were similar to the 240-items version with Cronbach’s alpha ranging in this sample from teamwork α = .61 to spirituality α = .90. The response format was a five-point scale from ‘strongly agree’ (= 5) to ‘strongly disagree’ (= 1). Item examples are: ‘I always keep my promises’ (honesty); ‘I am never too busy to help a friend’ (kindness); ‘I am always willing to take risks to establish a relationship’ (love).

#### Applicability of Character Strengths

The ‘Applicability of Character Strengths Rating Scales’ (ACS-RS; Harzer and Ruch 2013) were applied to evaluate the applicability of the five highest individual character strengths identified with the VIA-120 (also called signature character strengths according to Peterson and Seligman 2004) in regard to work and private life. For each of the top five individual signature character strength eight items (four questions referring to work and personal life each) were rated on a five-point scale from ‘never’ (= 1) to ‘(almost) always’ (= 5). The ACS-RS focus on the individual perception of four influences: two external (normative demands and appropriateness of strength-related behavior) and two internal (perceived presence of factors that may facilitate or restrain strength-related behavior and intrinsic motivation to show certain behavior; Harzer and Ruch 2013). Therefore, the items ask if the character strength is ‘demanded’, ‘helpful’, ‘important for me’, and ‘used’ in work or private life. The internal consistencies in this sample ranged from α = .80 to α = .89.

#### Well-Being

The German version of the ‘Comprehensive Inventory of Thriving’ (CIT; Hausler et al. 2017; original: Su et al. 2014) was used to measure different aspects of well-being. It comprises 54 items rated on a five-point scale ranging from ‘strongly disagree’ (= 1) to ‘strongly agree’ (= 5). The items measure 18 aspects of well-being, three assigned to subjective well-being (SWB) and fifteen to psychological well-being (PWB) as composite scores (see Hausler et al. 2017). SWB comprises life satisfaction, positive and negative emotions; PWB includes autonomy, engagement, mastery (accomplishment, learning, self-efficacy, self-worth, and skills), meaning, optimism and relationships (belonging, community, loneliness, respect, support, and trust). Each aspect is measured with three items. A general well-being mean score including all SWB and PWB aspects can be calculated as well. Cronbach’s α in this sample was .95 for SWB and .92 for PWB. Item examples are: ‘My life is going well’ (SWB; life satisfaction); ‘There are people I can depend on to help me’ (PWB; relationship).

#### Work Engagement

Work engagement was measured with the German nine-item short-version of the ‘Utrecht Work Engagement Scale’ (UWES; Schaufeli and Bakker 2003; Schaufeli et al. 2006) which is defined as a positive, fulfilling work-related state of mind that is characterized by vigor, dedication, and absorption (Schaufeli et al. 2006). The response format was a seven-point scale ranging from ‘never’ (= 0) to ‘always’ (= 6). Cronbach’s α in the present study was .94. An item example is: ‘At my job, I feel strong and vigorous’.

#### Burnout

For measuring the three dimensions of burnout, the adapted and modified German version of the ‘Maslach-Burnout-Inventory’ (MBI-D; Büssing and Perrar 1992) was used. It consists of 21 items comprising the three dimensions of emotional exhaustion (nine items), depersonalization (five items) and reduced personal accomplishment (seven items). They can be answered on a six-point scale from ‘never’ (= 0) to ‘very often’ (= 5). Satisfactory Cronbach’s α in this sample were found, ranging from .70 (depersonalization) to .91 (emotional exhaustion). Item examples are: ‘I feel emotionally drained from my work’; ‘I don’t really care what happens to some patients’; ‘I have accomplished many worthwhile things in this job’.

### Statistical Analyses

For all statistical analyses IBM SPSS Statistics 24 (IBM Corp. 2015) was used. Means ± standard deviation, minimum/maximum scores, skew, kurtosis and scale reliabilities were calculated to describe the metric properties of the items and the scales. Pearson’s coefficient inter-correlations were computed to assess the relationships of the study variables which can be interpreted as follows: *r* < .10 = no correlation, *r* = .10–.29 = low correlation, *r* = .30–.49 = moderate correlation, *r* ≥ .50 = high correlation (Cohen 1988). Cronbach’s α indicates acceptable internal consistency when values are >.70 (Nunnally 1978; see Peterson 1994). A priori G*power analyses were conducted, revealing a minimum of *N* = 74 to be powerful (one-tailed, alpha-error .05, statistical power .95, min. effect size 0.15; Faul et al. 2009). Hierarchical multiple linear regressions were performed (first step: VIA-120 signature character strength, method: enter; second step: applicability of signature character strengths in work (ASCS-W) and private life (ASCS-P), method: enter) to examine if respective applicabilities explained variance above and beyond the possession of the signature character strength. Their possession in model 1 and additionally their applicability in model 2 were the independent variables and subjective/psychological well-being, work engagement, and burnout (emotional exhaustion, depersonalization, reduced personal accomplishment) the dependent variables. ASCS-W was analyzed in all regressions (in regard to all outcomes), whereas ASCS-P was only analyzed in the regressions concerning SWB and PWB.

## Results

Descriptive statistics for all study variables including mean ± SD, min./max. scores, skew, kurtosis, and Cronbach’s α are presented in Table 1, ranging from α = .61 (teamwork) to α = .95 (subjective well-being). First, we identified the five most frequently reported individual signature character strengths in the whole sample being honesty (*N* = 144), followed by kindness (*N* = 137), love (*N* = 132), judgment (*N* = 131), and fairness (*N* = 92). The five character strengths most seldom among physicians’ individual signature character strengths were spirituality (*N* = 11), perspective (*N* = 14), self-regulation (*N* = 16), prudence (*N* = 18), and leadership (*N* = 20). Means of all character strengths (VIA-120: 1–5) ranged from spirituality (2.33 ± 0.95) to honesty (4.21 ± 0.44). Out of the five signature character strength applicability scores (ACS-RS: 1–5) love had both the lowest and highest rating at work (3.57 ± 0.91) and in private life (4.42 ± 0.68). Definitions of the most frequently reported signature character strengths of the sample can be found in the appendix (Table 5). In terms of well-being (CIT: 1–5) and work engagement (UWES: 0–6) means ranged roughly in the upper third (3.91 ± 0.70/3.84 ± 0.40; 3.66 ± 1.09), regarding burnout (MBI-D: 0–5) in the lower half (from 1.31 ± 0.50 to 2.36 ± 0.96).

According to the first aim of this analysis, Pearson’s coefficient inter-correlations between all relevant study variables (Table 2) suggested that the questionnaires VIA-120 and ACS-RS measure empirically distinct constructs, namely the possession and applicability of the signature character strengths honesty, kindness, love, judgment, and fairness in this sample. The highest correlation was found between the signature character strength love and its applicability in private life (*r* = .48), sharing about 23% of variance. Moreover, the inter-correlations between the scales of ASCS-P and ASCS-W were only low to moderate (from kindness *r* = .39 to judgment *r* = .23), sharing a maximum of about 15% variance, or even none (love *r* = .01).

According to the second aim, hierarchical multiple linear regressions revealed that the applicability of signature character strengths (ASCS) explained variance of the respective outcomes above and beyond their possession, but in different ways or to a different extent in the tested models. In the first step (model 1), each signature character strength was entered separately to test the weight of their possession relating to the outcome. In the second step (model 2), the respective applicability at work (ASCS-W) and in private life (ASCS-P) was entered as well to test if and how this impacts the weight of the possession revealed before (e.g., decrease, increase, or mediation) concerning the outcomes.

(a) The ASCS explained no variance of subjective well-being (SWB) above and beyond the possession of signature character strengths (*R^2^*-changes; Table 3). The possession of fairness, honesty, and kindness was significantly related to SWB in both models, whereas the regression testing love led to an overall significant model 2, with neither possession nor applicability being significant on its own (*p* total). However, in model 2 the applicability of judgment in private life mediated the relation of the signature character strength judgment on SWB, exceeding the significant *β*-coefficient from model 1 (Table 3).
(b) The ASCS explained variance of psychological well-being (PWB) above and beyond the possession of fairness, honesty, judgment, and love (*R^2^*-changes; Table 3). PWB was significantly related to the possession of all signature character strengths in both models, and to the applicability of fairness, honesty, judgment, and love in model 2. The applicability of honesty and love at work revealed similar β-coefficients compared to the possession β-coefficients in model 2, whereas different weights were found concerning the applicability in private life of fairness (applicability: β *=* .24; possession: β *=* .44) and judgment (applicability: β *=* .37; possession: β *=* .30). The applicability of kindness at work or in private life seemed not to be relevant for PWB in this sample.
(c) The ASCS explained variance of work engagement above and beyond the possession of fairness, honesty, judgment, and love (*R^2^*-changes; Table 4). Concerning honesty and love, only their applicability at work resulted in the significant *β*-coefficients (model 2) and moreover, the applicability of both fairness and judgment at work mediated the relation of the signature character strength itself and work engagement, exceeding the significant β-coefficients from model 1 (Table 4).
(d) The applicability of judgment at work revealed significant β-coefficients in emotional exhaustion and depersonalization, therefore significantly explaining variance above and beyond the possession of judgment in model 2 (*R^2^*-changes; Table 4). Reduced personal accomplishment was significantly related to the possession of judgment and kindness in both models, whereas the applicability of fairness at work mediated the relation of the signature character strength itself and reduced personal accomplishment, exceeding the significant β-coefficient from model 1 (Table 4).

## Discussion

The analysis of Austrian hospital physicians revealed that the questionnaires VIA-120 and ACS-RS measure empirically distinct constructs concerning the possession and applicability of the signature character strengths (ASCS) fairness, honesty, judgment, kindness, and love (shared variance <24%). All signature character strengths correlated significantly higher with applicability in private life (ASCS-P) than with applicability at work (ASCS-W). Hierarchical multiple linear regressions revealed that the ASCS explained variance of the respective outcomes above and beyond the possession of signature character strengths, but only in about one third of the tested models significantly. The applicability of fairness, honesty, judgment, and love explained additional variance for PWB (work: honesty and love; private life: fairness and judgment) and work engagement. In particular at work, the applicability of judgment explained additional variance for emotional exhaustion and depersonalization, as well did the applicability of fairness for reduced personal accomplishment.

The literature on (signature) character strengths research implies that in general their application rather than possession contributes to well-being (Govindji and Linley 2007; Rath 2007) but there has been little discussion of their applicability in daily life so far (Harzer and Ruch 2013). The environment and circumstances in which the behavior of a person usually occurs (e.g., opportunity to demonstrate behavior due to situational factors) is crucial and the importance of contextual factors in positive psychology has already been acknowledged in the past (Seligman and Csikszentmihalyi 2000). With the ACS-RS, the extent to which character strengths are applicable at work or in private life (Harzer and Ruch 2013) can be measured and considered separately. As all of the five most frequently reported signature character strengths (fairness, honesty, judgment, kindness, and love) correlated higher with applicability in private life than with applicability at work, the assumption arises that the degree of possessing (one of these) character strengths is more decisive in the relation to private life than to work. So a higher degree of possession correlated with more perceived applicability in private life, whereas in work life e.g., the degree of possessing honesty and love did not correlate with the respective applicability at all. Therefore, it was not relevant if physicians had lower or higher honesty or love scores relating to their applicability rating at work. Physicians are exposed to tremendous work demands and stressors (e.g., lack of autonomy, organizational stressors, adverse working hours), therefore kept busy with occupational challenges and not experiencing high applicability of honesty or love. This was further supported by the lowest scores of applicability of honesty and love at work. But if they experienced applicability, an additional contribution to PWB and work engagement revealed. Overall, physicians evaluated the applicability of characters strengths in private life significantly higher and maybe due to this they can compensate ‘missing’ applicability of character strengths at work. There could be other reasons as well as external factors which might have more or additional constraining influence on ASCS-W (e.g., no necessity or desirability at work, personal expectation to behave in a different way, restraining organizational structures).

External factors can also have a supporting effect, e.g., the socio-moral climate in an organization. It is characterized by discursive, participative, appreciative, supportive, and caring interactions between supervisors, subordinates, and co-workers (Pircher Verdorfer et al. 2012; Weber et al. 2008). As a social climate allows or even encourages employees to express their individual perspectives, opinions, and standpoints (independently of their rank or formal position), it is conceivable that it positively correlates with ASCS-W. In this regard first evidence has been given, even for a reciprocal relation (impact of socio-moral climate on the ASCS six months later, and an even stronger reversed effect; Höge et al. 2018, in this special issue). Other work characteristics like skill adequacy, cognitive (challenging) demands, or autonomy could also play an important role in terms of personality development (Glaser et al. 2015), further positively influencing ASCS-W. They could be seen as ‘catalyzers’ enabling individuals to apply their signature character strengths. The fit between a person and the work task (skill adequacy) would be a highly advisable example (e.g., Harzer and Ruch 2013). Otherwise the employee is concerned with excessive or unchallenging demands and may lose the ability to develop optimally. Cognitive demands and autonomy can further push intrinsic motivation and as individuals are primarily intrinsically motivated to apply their strengths (Linley et al. 2010) their existence at work must be considered in regard to their ASCS-W. A recent study revealed that these work characteristics in hospitals are significantly associated with physicians’ ASCS (Strecker et al. 2018, in this special issue). Therefore, attempts to strengthen well-being at work by means of applying signature character strengths in the future should definitely take work characteristics into account, develop strategies to manage workload efficiently, and promote physicians’ abilities to increase resources for successful coping.

The most frequently reported signature character strengths of the participating physicians in this sample (honesty, kindness, love, judgment, and fairness) differed in their patterns in regard to the respective outcomes. These different patterns might partially be explained by the definition of the character strengths themselves (see appendix Table 5), their required conditions for applicability as well as by the corresponding outcome. For example, possessing kindness was significantly related to SWB, PWB, and reduced personal accomplishment. Kindness can be numbered among the so-called ‘tonic’ strengths, being applicable in more situations than ‘phasic’ strengths, being relevant for specific situations only (Peterson and Seligman 2004). Therefore, high applicability might not be that crucial for being kind in work or private life across many situations, whereas judgment can rather be counted a ‘phasic’ strength, definitely requiring more applicability. This was supported by significant results of ASCS-P where perceived applicability seemed to be relevant for well-being (SWB/PWB) and even more for work with situational factors calling for the demonstration of judgment (work engagement, emotional exhaustion, and depersonalization). Emotional exhaustion and depersonalization were in particular significantly negative related to the applicability of judgment at work. Therefore, having the time and opportunity to think critically, considering decisions carefully without mental pressure, and weighing all evidence fairly can protect physicians who possess the signature character strength against emotional exhaustion and depersonalization, being the two key dimensions of burnout (Leiter and Maslach 2016). Moreover, the applicability of fairness at work was significantly related to reduced personal accomplishment the same way, so that the possibility of treating all people the same and giving everyone a fair chance despite work demands or stressors seemed to strengthen perceived effectiveness in the hospital.

The absence of burnout symptoms does not automatically mean that a person is engaged in the work tasks. Although job burnout and work engagement are strongly negatively related, factor analytical results indicate that they are distinctive (Schaufeli et al. 2002). Moreover, outlining the Job-Demands-Resources-Model of Engagement and Burnout, Bakker and Demerouti (2007) argued that both constructs are triggered by different underlying processes. Burnout is predominantly caused by work demands (e.g., workload, work stressors) whereas work engagement is primarily fueled by task-related and personal resources (e.g., job control, autonomy, task variability, social support, or self-efficacy beliefs). As character strengths can be understood as personal resource, the importance of being applicable at work is evident. Therefore, perceived applicability of fairness, honesty, judgment, and love was essential for hospital physicians’ work engagement and moreover, as well for their PWB (honesty and love at work, fairness and judgment in private life). Beside their applicability, their possession (including kindness) was important as well, suggesting that both constructs have a strong relationship to PWB, even stronger than to SWB (no tested model including the ASCS was significantly adding incremental variance). However, the applicability of judgment in private life mediated the relation of judgment and SWB (‘phasic’ strength) and possessing fairness, honesty, or kindness was an indicator for a significant positive relation with SWB. Previous results already suggested that the overall ‘good character’ (Hausler et al. 2017a) as well as the applicability at work (Hausler et al. 2017b) was significantly more closely related to PWB than to SWB. Therefore, one could interpret the existing results as supportive for the hypothesis of PWB and signature character strengths having a stronger reciprocal relationship already due to their respective definitions, in contrast to emotions and satisfaction in different life domains.

### Implications

Based on the presented analysis, there is a difference between the possession and the applicability of fairness, honesty, judgment, kindness, and love at work and in private life. Further studies should take that into account together with the difference of tonic/phasic strengths and the influence of setting variables (e.g., demands/stressors; sociomoral climate; fit between person and surrounding). Caution with labeling the constructs is encouraged whether it is a question of possessing or applying character strengths. Practically, creating awareness for individual signature character strengths as well as focusing on the setting variables to enhance the applicability in hospitals could be a starting point to improve physicians’ well-being. This would be in line with the third pillar of positive psychology by developing positive institutions. Although diverse patterns were found, all of the signature character strengths added something to physicians’ well-being. In particular the applicability of fairness and judgment took a mediating role substantiating further experimental research to explicitly test if more applicability of character strengths consistent with organizational structures has the potential to enhance physicians’ well-being. Moreover, supervisors could be informed of their importance and trained to ‘demand’ for strengths or to evaluate them as ‘helpful’. Honesty, love and kindness (and their applicability) could be fostered in terms of being ‘important’ in particular for jobs involving other people.

## Limitations and Future Research Suggestions

Presented cross-sectional data are limited to results from one occupational group from two hospitals based upon subjective assessment only. Therefore, country-specific work structures, organizational procedures or personal requirements may have influenced the perception of applicability in a certain way to which signature character strengths are ‘demanded’, ‘helpful’, or ‘important’ in work life, raising the question how these ratings would look like in other hospitals. Moreover, all self-ratings could contain effects of self-representation or social desirability relating to the medical profession as well as other response biases. In particular when examining SWB, assimilation (valuative judgments towards the position of context stimuli) or contrast effects (negative correlation between a judgment and contextual information) can occur (Schwarz and Strack 1999). Moreover, the reduced sample size needs to be considered when interpreting the data concerning the signature character strength fairness. Finally, to give causal inter-pretations and generalized implications, longitudinal studies including more hospitals and peer-ratings with different organizational structures are needed, further including different cultures or behavioral/objective measures (e.g., social perception of signature character strengths from related party; observation of acting in real situations).

Furthermore, physicians only received feedback on their five highest ranked character strengths from the VIA-120 and so the ASC-RS were only answered for these respectively. As other patterns could occur regarding the applicability of other signature character strengths, future study designs might profit by analyzing the applicability of all character strengths in order to get a more profound understanding concerning different domains. The patterns discovered in this analysis are differing and therefore not directly transferable to other signature character strengths. Future research could also gain deeper insights when additionally analyzing more differentiated domains beside ‘work’ and ‘private life’ according to the various life domains in SWB to be considered (e.g., leisure, health, finances, one’s group; Diener et al. 1999).

Beside the ASCS, other individual strengths focusing on e.g., occupational settings (Buckingham and Clifton 2001) or a comprehensive applicability at work and in various life domains (Linley and Harrington 2006) could significantly influence well-being, work engagement, and burnout positively as well. These concepts define strengths as natural capacities coming from within that one yearns to use, that enable authentic expression and that energize (Govindji and Linley 2007), and which belong to positive traits or psychological capacities/talents refined with knowledge and skills (Proctor et al. 2011). This definition comprises character strengths, but is not limited to them, therefore including e.g., sportiness, manual skills, cooking, health maintaining strategies, multicultural competence, peer resistance, perfectionism, ability to relax, amusement, organizational abilities, optimistic thinking, etc. Their application was positively related to well-being (e.g., Huber et al. 2017), but their applicability in terms of being ‘demanded’, ‘helpful’, or ‘important’ has not been analyzed explicitly. As the applicability seems to be an empirically distinct construct according to the results from this analysis, the ACS-RS items could be ‘translated’ into a future instrument examining the applicability of individual strengths in different domains.

## Conclusions

Data led to the assumption that the possession as well as the applicability of signature character strengths (fairness, honesty, judgment, kindness, or love) at work and in private life was important, but to differing degrees. Possessing the signature character strengths of fairness, honesty, or kindness - but not their applicability - was an indicator for a significant positive relation with subjective well-being as well as only the possession of judgment and kindness seemed to interact negatively with reduced personal accomplishment. However, hospital physicians’ applicability of signature character strengths fairness, honesty, judgment, and love was particularly essential for their psychological well-being and work engagement, more than their possession. Concerning the dimensions of burnout, only the applicability of judgment (emotional exhaustion, depersonalization) and fairness (reduced personal accomplishment) seemed to play a role. Overall, the analyses revealed diverse patterns for fairness, honesty, judgment, kindness, and love of Austrian hospital physicians, allowing first statements on their meaning.

## Figures and Tables

**Table 1:** Descriptives and Cronbach’s α for all study variables (*N* = 274)

	mean (SD)	min. | max.	skew | kurtosis	α
VIA-120				
(1) Wisdom and knowledge				
Creativity	3.45 (.69)	1.00 | 5.00	−0.25 | 0.35	.85
Curiosity	3.84 (.55)	2.20 | 5.00	−0.37 | 0.06	.72
Judgment	4.00 (.51)	2.20 | 5.00	−0.20 | −0.09	.73
Love of learning	3.64 (.68)	1.60 | 5.00	-0.09 | -0.29	.76
Perspective	3.37 (.53)	2.00 | 5.00	0.06 | 0.21	.65
(2) Courage				
Bravery	3.43 (.63)	1.20 | 5.00	−0.15 | 0.10	.74
Honesty	4.21 (.44)	2.80 | 5.00	−0.27 | -0.20	.62
Perseverance	3.93 (.58)	2.00 | 5.00	−0.55 | 0.48	.75
Zest	3.49 (.65)	1.80 | 5.00	−0.21 | -0.25	.78
(3) Humanity				
Kindness	4.10 (.50)	2.80 | 5.00	−0.18 | -0.27	.73
Love	4.03 (.67)	1.60 | 5.00	−0.82 | 0.90	.78
Social intelligence	3.89 (.52)	2.40 | 5.00	−0.30 | -0.08	.70
(4) Justice				
Fairness	4.03 (.55)	2.00 | 5.00	−0.68 | 0.61	.73
Leadership	3.66 (.53)	2.00 | 5.00	0.06 | -0.05	.66
Teamwork	3.71 (.50)	2.00 | 5.00	−0.31 | 0.54	.61
(5) Temperance				
Forgiveness	3.44 (.63)	1.80 | 5.00	−0.05 | -0.20	.66
Humility	3.29 (.63)	1.40 | 4.80	−0.10 | 0.01	.69
Prudence	3.49 (.60)	1.80 | 4.80	−0.16 | -0.24	.71
Self-regulation	3.15 (.69)	1.20 | 4.80	−0.19 | -0.14	.65
(6) Transcendence				
Appreciation of beauty & excellence	3.51 (.66)	1.60 | 5.00	−0.11 | -0.50	.74
Gratitude	3.53 (.62)	1.80 | 5.00	0.07 | -0.25	.77
Hope	3.71 (.60)	2.00 | 5.00	−0.31 | -0.01	.68
Humor	3.71 (.68)	2.00 | 5.00	−0.11 | -0.37	.82
Spirituality	2.33 (.95)	1.00 | 5.00	0.56 | -0.33	.90
ASCS-W (Top 5)				
Fairness	4.00 (.70)	2.50 | 5.00	−0.30 | -0.69	.75
Honesty	3.96 (.76)	2.00 | 5.00	−0.46 | -0.45	.80
Judgment	4.04 (.70)	2.00 | 5.00	−0.51 | 0.14	.75
Kindness	4.07 (.69)	2.25 | 5.00	−0.35 | -0.72	.69
Love	3.57 (.91)	1.00 | 5.00	−0.41 | -0.27	.83
ASCS-P (Top 5)				
Fairness	4.26 (.60)	3.00 | 5.00	−0.39 | -0.63	.82
Honesty	4.33 (.65)	2.00 | 5.00	−0.96 | 0.83	.86
Judgment	4.17 (.67)	2.25 | 5.00	−0.41 | -0.45	.82
Kindness	4.27 (.68)	2.00 | 5.00	−0.93 | 0.76	.81
Love	4.42 (.68)	2.00 | 5.00	−1.30 | 1.45	.91
CIT				
Subjective well-being	3.91 (.70)	1.33 | 5.00	−0.83 | 0.89	.95
Psychological well-being	3.84 (.40)	2.58 | 4.76	−0.47 | 0.34	.92
UWES				
Work Engagement	3.66 (1.09)	.22 | 5.78	−0.47 | -0.21	.94
MBI-D				
Emotional exhaustion	2.36 (.96)	.11 | 4.67	0.44 | -0.17	.91
Depersonalization	1.45 (.78)	.00 | 4.20	0.32 | -0.33	.70
Reduced personal accomplishment	1.31 (.50)	.00 | 3.00	0.02 | 0.27	.73

ASCS-P/-W = applicability of signature character strengths in private life/at work, CIT = Comprehensive Inventory of Thriving, MBI-D = Maslach Burnout Inventory-Deutsch, SD = standard deviation, UWES = Utrecht Work Engagement Scale, VIA-120 = Values in Action-Inventory of Strengths

**Table 2:** Inter-correlations for the possession of character strengths (VIA-120) and their applicability at work (ASCS-W) and in private life (ASCS-P), subjective/psychological well-being (SWB/PWB), work engagement (WE), and burnout (EE: emotional exhaustion; DP: depersonalization; RPA: reduced personal accomplishment) and the respective number of participants in the opposite cell

		1	2	3	4	5	6	7	8	9	10	11	12	13	14	15	16	17	18	19	20	21
	VIA-120																					
1	Fairness	–	274	274	274	274	65	102	85	91	91	67	102	86	92	92	217	217	222	218	218	218
2	Honesty	.49**	–	274	274	274	65	102	85	91	91	67	102	86	92	92	217	217	222	218	218	218
3	Judgment	.27**	.34**	–	274	274	65	102	85	91	91	67	102	86	92	92	217	217	222	218	218	218
4	Kindness	.50**	.45**	.19**	–	274	65	102	85	91	91	67	102	86	92	92	217	217	222	218	218	218
5	Love	.27**	.36**	.17**	.34**	–	65	102	85	91	91	67	102	86	92	92	217	217	222	218	218	218
	ASCS-W																					
6	Fairness	.35**	.14	.27*	.19	.31*	–	42	24	26	23	65	42	24	26	23	55	55	56	55	55	55
7	Honesty	.09	.10	.16	–.05	.02	.43**	–	36	49	52	42	102	36	49	52	91	91	93	89	89	89
8	Judgment	.13	.25*	.30**	.03	–.04	.16	.19	–	37	36	24	36	85	37	36	75	75	76	74	74	74
9	Kindness	.01	.09	.18	.23*	.27**	.57**	.42**	.39**	–	46	26	49	37	91	46	75	75	80	77	77	77
10	Love	.09	.14	.09	.13	–.03	.07	.17	.32	.02	–	23	52	36	46	91	76	76	79	76	76	76
	ASCS-P																					
11	Fairness	.46**	.58**	.39**	.35**	.38**	.26*	.36*	.11	.39*	.66**	–	42	25	27	24	55	55	56	55	55	55
12	Honesty	.06	.36**	.13	.16	.29**	.17	.36*	.22	.19	.25	.62**	–	36	49	52	91	91	93	89	89	89
13	Judgment	.17	.30**	.33**	.08	.25**	.03	.01	.23*	.25	.41*	.29	.34*	–	38	36	75	75	76	74	74	74
14	Kindness	.13	.36**	.33**	.44**	.48**	.03	.05	.39*	.39**	–.24	.37	.41**	.49**	–	46	75	75	80	77	77	77
15	Love	.14	.14	.09	.22*	.48**	–.08	.08	.12	.10	.01	.12	.04	.10	.59**	–	76	76	79	76	76	76
16	SWB	.16*	.24**	.06	.15*	.42**	.17	.18	.02	.25*	.16	.30*	.26*	.32	.24*	.27*	–	217	217	214	214	214
17	PWB	.23**	.34**	.18**	.26**	.49**	.34*	.38**	.15	.33**	.24	.47**	.36**	.46**	.29*	.24*	.73**	–	217	214	214	214
18	WE	.10	.14*	.14*	.13*	.17*	.39**	.32	.28*	.20	.40**	.30*	.18	.25*	.13	.06	.51**	.56**	–	218	218	218
19	EE	.02	.04	.03	.07	–.17*	–.24	–.17	–.25*	–.22	–.09	–.09	–.23*	–.08	–.16	–.24*	–.50**	–.43**	–.57**	–	218	218
20	DP	–.23**	–.21**	–.01	–.01	–.04	–.17	–.15	–.30**	–.12	–.04	–.11	–.11	–.15	–.19	–.04	–.18**	–.23**	–.28**	.41**	–	218
21	RPA	–.19**	–.27**	–.15*	–.15*	–.10	–.33*	–.22*	–.23	–.17	–.20	–.30*	–.13	–.29*	–.12	–.11	–.25**	–.44**	–.49**	.28**	.38**	–

*p < .05, ** *p* < .01 (two-tailed)

**Table 3:** Hierarchical multiple linear regressions including the applicability of signature character strengths in work (ASCS-W) and private life (ASCS-P) into the possession (VIA-120) and subjective/psychological well-being (SWB / PWB) relation

Outcome: SWB		Model 1		Model 2				
		*R^2^*	*β*	*R^2^*	*R^2^*-change	*β*	*p* total	*N*
Fairness	VIA-IS 120	.205	.453**	.221	.016	.390**	**.005**	91
	ASCS-W					.053		
	ASCS-P					.118		
Honesty	VIA-IS 120	.093	.305**	.130	.037	.251*	**.007**	75
	ASCS-W					.102		
	ASCS-P					.139		
Judgment	VIA-IS 120	.054	.232*	.128	.074	.168	**.020**	76
	ASCS-W					–.089		
	ASCS-P					.287*		
Kindness	VIA-IS 120	.105	.324**	.142	.037	.260*	**.012**	75
	ASCS-W					.164		
	ASCS-P					.070		
Love	VIA-IS 120	.047	.217	.113	.066	.119	**.034**	55
	ASCS-W					.177		
	ASCS-P					.215		
Outcome: **PWB**								
Fairness	VIA-IS 120	.344	.586***	.438	.094*	.440***	**<.001**	91
	ASCS-W					.188		
	ASCS-P					.243*		
Honesty	VIA-IS 120	.135	.368***	.279	.143***	.288**	**<.001**	75
	ASCS-W					.295**		
	ASCS-P					.166		
Judgment	VIA-IS 120	.162	.403***	.286	.123**	.295**	**<.001**	76
	ASCS-W					–.013		
	ASCS-P					.370**		
Kindness	VIA-IS 120	.167	.408***	.230	.063	.329**	**<.001**	75
	ASCS-W					.227		
	ASCS-P					.071		
Love	VIA-IS 120	.099	.314**	.177	.078*	.273*	**.003**	55
	ASCS-W					.263*		
	ASCS-P					.111		

Notes: **bold** = significant; *p*-values for β, *R^2^* -changes and total = * p < .05, ** p < .01, *** *p* < .001; *p* total values = significance of model 2 in total (possession and applicability in work & private life taken into account), N = number of participants

**Table 4:** Hierarchical multiple linear regressions including the applicability of signature character strengths at work (ASCS-W) into the possession (VIA-120) and work engagement (WE), emotional exhaustion (EE), depersonalization (DP) and reduced personal accomplishment (RPA) relation

Outcome: WE		Model 1		Model 2				
		*R^2^*	*β*	*R^2^*	*R^2^*-Change	*β*	*p* total	*N*
Fairness	VIA-120	.105	.324*	.213	.108**	.250	**.002**	93
	ASCS-W					.337**		
Honesty	VIA-120	.020	.142	.120	.100**	.126	**.003**	80
	ASCS-W					.317**		
Judgment	VIA-120	.052	.229*	.103	.051*	.171	**.019**	79
	ASCS-W					.233*		
Kindness	VIA-120	.043	.208	.069	.026	.173	.064	76
	ASCS-W					.164		
Love	VIA-120	<.001	.009	.163	.163***	.034	**.001**	56
	ASCS-W					.404***		
Outcome: **EE**								
Fairness	VIA-120	.003	−.057	.056	.053	−.004	.225	89
	ASCS-W					−.235		
Honesty	VIA-120	.001	.032	.029	.028	.044	.282	77
	ASCS-W					−.168		
Judgment	VIA-120	<.001	.017	.072	.072*	.089	.071	76
	ASCS-W					−.277*		
Kindness	VIA-120	<.001	−.011	.048	.048	.039	.163	74
	ASCS-W					−.224		
Love	VIA-120	.002	−.048	.010	.008	−.052	.688	55
	ASCS-W					−.089		
Outcome: **DP**								
Fairness	VIA-120	.001	−.029	.029	.028	.010	.464	89
	ASCS-W					−.173		
Honesty	VIA-120	.002	−.042	.024	.022	−.031	.358	77
	ASCS-W					−.148		
Judgment	VIA-120	.009	−.095	.089	.080*	−.018	**.037**	76
	ASCS-W					−.293*		
Kindness	VIA-120	.025	−.159	.033	.008	−.138	.284	74
	ASCS-W					−.093		
Love	VIA-120	.002	.041	.003	.001	.039	.898	55
	ASCS-W					−.036		
Outcome: **RPA**								
Fairness	VIA-120	.079	−.281*	.155	.077*	−.217	**.012**	89
	ASCS-W					−.284*		
Honesty	VIA-120	.026	−.160	.068	.042	−.146	**.048**	77
	ASCS-W					−.206		
Judgment	VIA-120	.143	−.378**	.161	.018	−.343**	**.002**	76
	ASCS-W					−.138		
Kindness	VIA-120	.104	−.322**	.115	.011	−.298*	.011	74
	ASCS-W					−.107		
Love	VIA-120	.006	−.078	.046	.040	−.089	.178	55
	ASCS-W					−.201		

Notes: **bold** = significant; *p*-values for β, *R^2^* -changes and total = * p < .05, ** p < .01, *** p < .001; *p* total values = significance of model 2 in total (possession and applicability in work life taken into account), N = number of participants

## Appendix

**Table 5:** Definitions of the top-five signature character strengths (Peterson and Seligman 2004)

Character strength	Definition
Fairness	Treating all people the same according to notions of fairness and justice; not letting personal feelings bias decisions about others; giving everyone a fair chance
Honesty	[integrity, authenticity]: Speaking the truth but more broadly presenting oneself in a genuine way and acting in a sincere way; being without pretense; taking responsibility for one’s feelings and actions
Judgment	[open-mindedness, critical thinking]: Thinking things through and examining them from all sides; not jumping to conclusions; being able to change one’s mind in light of evidence; weighing all evidence fairly
Kindness	[generosity, nurturance, care, compassion, altruistic love, “niceness”]: Doing favors and good deeds for others; helping them; taking care of them
Love	Valuing close relations with others, in particular those in which sharing and caring are reciprocated; being close to people
